# Increased delay to lung transplantation for women candidates: gender-based disparity matters in the lung transplant trajectory

**DOI:** 10.1183/23120541.00623-2024

**Published:** 2025-05-06

**Authors:** Adrien Tissot, Anne-Sophie Coatanea, Olivia Rousseau, Antoine Roux, Benjamin Coiffard, Xavier Demant, Benjamin Renaud-Picard, Jérôme Le Pavec, Antoine Magnan, Jean-François Mornex, Thomas Villeneuve, Loïc Falque, Mathilde Salpin, Véronique Boussaud, Christiane Knoop, Martine Reynaud-Gaubert, Romain Kessler, Gaëlle Dauriat, David Lair, Aurore Foureau, François-Xavier Blanc, Mathilde Karakachoff, Patricia Lemarchand, the COLT consortium

**Affiliations:** 1 Nantes Université, CHU Nantes, INSERM, Service de Pneumologie, L'Institut du Thorax, Center for Research in Transplantation and Translational Immunology, UMR 1064, Nantes, France; 2 Nantes Université, CHU Nantes, Service de Pneumologie, L'Institut du Thorax, Nantes, France; 3 Nantes Université, CHU Nantes, Pôle Hospitalo-Universitaire 11: Santé Publique, Clinique des Données, INSERM, CIC 1413, Nantes, France; 4 Pneumology, Adult Cystic Fibrosis Center and Lung Transplantation Department, Hôpital Foch, Université de Versailles Saint Quentin Paris-Saclay, INRAe UMR 0892, Paris, France; 5 Paris Transplant Group, Paris, France; 6 Aix Marseille Université, Department of Respiratory Medicine and Lung Transplantation, AP-HM, Hôpital Nord, Marseille, France; 7 Service de Pneumologie, CHU Bordeaux, Pessac, France; 8 Service de Pneumologie, Hôpitaux Universitaires de Strasbourg, Strasbourg, France; 9 Service de Pneumologie et Transplantation Pulmonaire, Groupe Hospitalier Marie-Lannelongue – Saint Joseph, Université Paris-Saclay, Le Kremlin-Bicêtre, UMR_S 999, Université Paris-Sud, INSERM, Paris, France; 10 Université de Lyon, Université Lyon 1, PSL, EPHE, INRAE, IVPC, Hospices Civils de Lyon, Groupement Hospitalier Est, Service de Pneumologie, Orphalung, RESPIFIL Lyon, Lyon, France; 11 Service de Pneumologie, Centre de Compétence des Maladies Pulmonaires Rares, Hôpital Larrey, CHU Toulouse, Toulouse, France; 12 Service Hospitalier Universitaire de Pneumologie et Physiologie, Pôle Thorax et Vaisseaux, CHU Grenoble Alpes, Grenoble, France; 13 AP-HP Nord-Université Paris Cité, Hôpital Bichat, Service de Pneumologie B et Transplantation Pulmonaire, Université Paris Cité, PHERE UMRS 1152, Paris, France; 14 Service de Pneumologie, Hôpital Cochin, Paris, France; 15 CHU Erasme, Department of Chest Medicine, Brussels, Belgium; 16 Nantes Université, CHU Nantes, CNRS, INSERM, L'Institut du Thorax, Nantes, France

## Abstract

**Background:**

Lung transplantation is a highly dynamic segment of solid organ transplantation in which gender plays a central role. Our objective was to investigate the causes of outcome differences between women and men all along the lung transplantation pathway.

**Methods:**

We used data from the French COhort in Lung Transplantation (COLT) study (12 participating lung transplantation centres). Analyses were performed in three phases: baseline clinical characteristics, peri-transplantation period and post-transplantation follow-up.

**Results:**

Overall, 1710 participants (802 women and 908 men) were included in this study. Women were less likely than men to undergo transplantation (91.6% *versus* 95.6%; p=0.001) and waited longer before transplantation (115 *versus* 73 days; p<0.001). Female gender and pre-transplantation class I anti-human leukocyte antigen antibodies were identified as independent factors associated with longer waiting time duration. Female transplant recipients commonly received lungs from height- and sex-matched donors, despite higher female waiting list mortality and a higher proportion of male donors. Importantly, women with oversized lung transplantation (defined by predicted total lung capacity (pTLC) ratio and weight mismatch) did not have worse survival. The overall post-transplantation survival of female recipients was significantly higher than that of male recipients (65.6% *versus* 57.3%; p<0.001), although the prevalence of specific major lung transplantation outcomes did not differ according to gender.

**Conclusion:**

Women waited longer and were less likely to undergo transplantation. Women transplanted with an oversized lung did not have worse survival after transplantation, suggesting that size matching criteria based on pTLC ratio and weight mismatch may be less stringent in this context.

## Shareable abstract

**Women are less likely than men to get a lung transplant, and wait 6 weeks longer. Immunological factors and donor–recipient size matching are the main reasons. Post-transplantation survival was higher for women (70% alive at 5 years *versus* 61% of men).** https://bit.ly/4hrDxru

## Introduction

The number of lung transplantations performed each year has almost constantly risen since the 1990s, and to date nearly 70 000 adult procedures have been reported worldwide [1]. The field of lung transplantation has made significant advances over the last decades with improvement of donor lung utilisation and allocation [2–5], surgical techniques and intensive care management [6]. It is the fastest growing segment of organ transplantation, but despite those favourable trends, lung transplantation remains fraught with a high mortality risk and morbidity [7]. Understanding factors associated to the natural history of lung transplantation, from waiting list to short- and long-term survival, is important for predicting and potentially improving outcomes.

Gender as a critical intrinsic and extrinsic factor plays a pivotal role in the field of transplantation [8]. Recent studies have pointed out gender differences and disparities all along the transplantation pathway, from waiting list access to post-transplantation outcomes [9]. Lung transplantation data from the USA demonstrated that women had a lower chance of being transplanted than men (83.9% *versus* 88.7%) and that women waited longer before transplantation [10, 11]. As for survival after lung transplantation, there seems to be an advantage for female recipients which has not been fully explained [12, 13]. These studies have mostly been performed using the United Network of Organ Sharing (UNOS) registry and more studies from different countries in other regions of the world are needed to confirm their results. Furthermore, no study has attempted to specifically evaluate the potential differences between men and women both before and after transplantation in the same cohort, and to investigate the potential causes for these differences.

We performed a retrospective study of French participants extracted from the COhort in Lung Transplantation (COLT) database in order to carry out a comparative analysis between women and men at three different periods of the lung transplantation process: waiting list, lung transplantation itself and post-transplantation.

## Materials and methods

### Patients

Patients were selected among those enrolled in COLT (ClinicalTrials.gov: NCT00980967) [14]. The COLT cohort was a prospective study that included French patients between September 2009 and December 2018. The ethics committee (Comité de Protection des Personnes Ouest 1-Tours, 2009-A00036-51) approved the study and all participants provided written informed consent. To obtain a complete donor dataset, we selected patients who were also included in the French Biomedical Agency database [15]. Consequently, patients on the transplant waiting list of 12 French COLT partner centres were included. Patients with a previous history of lung transplantation were excluded. This study complied with the International Society for Heart and Lung Transplantation (ISHLT) statement on transplant ethics [16].

### Collected data

Patients were enrolled at the time of registration on the waiting list before lung transplantation and then followed up to 10 years post-transplantation. Clinical data were collected from a dedicated database approved by the Commission Nationale Informatique et Libertés (CNIL; authorisation number: 911142). Collected data included baseline characteristics such as demographics, medical history, pulmonary function tests (PFTs), transplantation data (surgery, pre- and post-operative complications) and follow-up outcomes (rejections, infections, treatment and PFT results). Gender was self-reported. The 6-min walk test was performed according to American Thoracic Society guidelines [17]. The following classifications were used to describe the underlying diseases: cystic fibrosis (CF), COPD/emphysema, pulmonary arterial hypertension, interstitial lung disease (ILD) (including idiopathic pulmonary fibrosis) and other (including scleroderma, sarcoidosis, lymphangioleiomyomatosis and other connective tissue diseases).

Chronic lung allograft dysfunction (CLAD) phenotype was assessed for every patient at 3 and 5 years post-transplantation or at death/re-transplantation by an adjudication committee. Classification was initially made according to the proposition of Verleden *et al*. [18] and updated from the 2019 ISHLT consensus report [19].

The French lung allocation policy is detailed in the supplementary material. The choice of a lung donor and matching with a recipient, including lung volume matching, was the responsibility of the local lung transplantation team with no written common policy. Weight mismatch was calculated as donor weight minus recipient weight. For the donor-to-recipient predicted total lung capacity (pTLC) ratio, we used donor and recipient pTLCs calculated according to the Global Lung Function Initiative references values [20, 21]. To address the non-linear association between pTLC ratio and overall survival, a spline was used to separate transplanted individuals into three relevant categories (supplementary figure S3) [22].

### Statistical analysis

Analysis were performed in three phases according to the transplantation process. The first set of results analysed the sociodemographic, medical history and baseline clinical characteristics. The second set analysed peri-transplantation period variables, including recipient–donor and mismatch characteristics. The third set of analyses involved the post-transplantation follow-up and graft rejection features.

The characteristics of women and men included in the study were expressed as number, median and proportion, according to the type of variable. Continuous variables were reported as mean with standard deviation or median (interquartile range (IQR)), and comparisons between groups were computed using the t-test in case of variables normally distributed and Wilcoxon's test otherwise. Categorical variables were reported as number (percentage) and comparisons were computed through Pearson's Chi-squared test or Fisher's test. Censored time-dependent variables, including living status, delay to transplantation, CLAD occurrence, high emergency transplantation and related time-to-event curves, were analysed using the Kaplan–Meier method. Log-rank tests were used to compare curves between genders, weight mismatch, gender-related mismatch, age class and other relevant groups.

Survival curve adjustment and the influence of predictor variables were investigated using Cox proportional hazards regression models after validation of the proportional hazard assumption. Hazard ratios (HRs), 95% confidence intervals and p-values were computed at univariable, gender-adjusted and multivariable levels. In gender-adjusted analysis, p-values were corrected following the Benjamini–Hochberg method. Variables presenting a p-value <0.2 in the univariable analysis were selected to perform a multivariable model using a stepwise forward variable selection with Akaike Information Criterion threshold.

Missing values were reported for each variable if applicable and were not included when performing comparison tests. No imputation was applied to missing data, excepted for medical history variables, considered as “not having the disease”, when missing. p<0.05 was considered as statistically significant. All analyses were performed using R version 4.0.4 (www.r-project.org).

## Results

### Pre-transplantation characteristics

**FIGURE 1:**
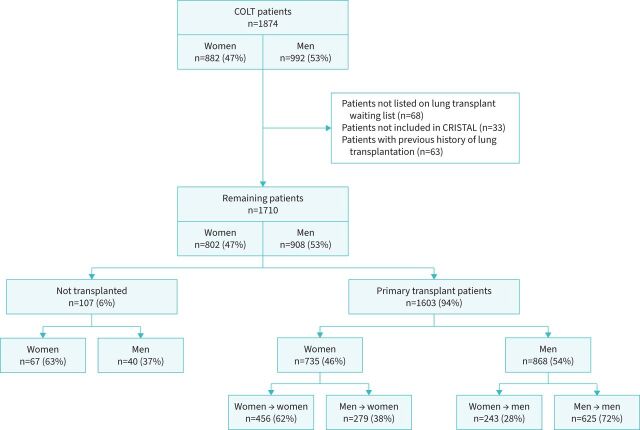
Patient flowchart. COLT: COhort in Lung Transplantation; CRISTAL: French Biomedical Agency database.

**FIGURE 2:**
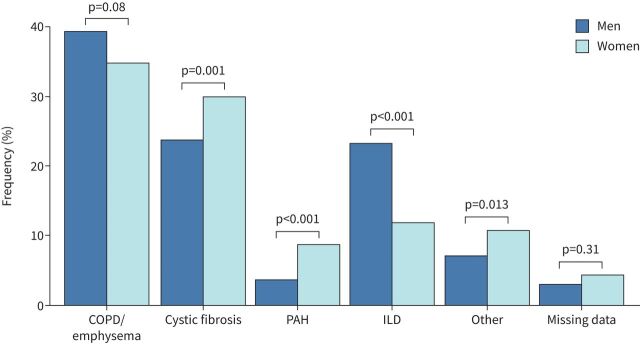
Underlying diagnosis in women and men awaiting lung transplantation. PAH: pulmonary arterial hypertension; ILD: interstitial lung disease. Chi-squared test used.

A total number of 1710 patients were included in the present study, of which 802 were women (47%) and 908 were men (53%) (figure 1). In both genders, the main underlying disease was COPD/emphysema, followed by CF and ILD (figure 2). At the time of registration on the lung transplant waiting list, women were younger (47 *versus* 54 years; p<0.001) (table 1) and displayed fewer comorbidities than men, including ischaemic heart disease (2.7% *versus* 6.8%; p<0.001) and cardiovascular risk factors (dyslipidaemia, high blood pressure and smoking history) (table 1). Mean height and weight were higher in men (67 *versus* 53 kg; p<0.001 and 173 *versus* 160 cm; p<0.001, respectively). Pre-transplantation anti-human leukocyte antigen (HLA) antibodies were more prevalent in women than in men (46% *versus* 26%; p<0.001) for both class I and II. Regarding respiratory failure severity, women were less likely to be on long-term oxygen therapy (81% *versus* 86%; p=0.03) and performed a slightly higher predicted distance in the 6-min walk test (45% *versus* 43% of predicted; p=0.03). PFTs according to the underlying disease are shown in supplementary table S1.

**TABLE 1:** Demographic and clinical characteristics in women and men listed for lung transplantation

	Women (n=802)	Men (n=908)	p-value
**Age (years)**	47 (32–57)	54 (39–60)	<0.001
**Medical history**			
Ischaemic heart disease	22 (2.7)	62 (6.8)	<0.001
Dyslipidaemia	36 (4.5)	64 (7.0)	0.032
High blood pressure	76 (9.5)	117 (12.9)	0.032
Diabetes	113 (14.1)	147 (16.2)	0.25
Heart failure	9 (1.1)	12 (1.3)	0.88
PH group III	89 (11.1)	105 (11.6)	0.82
Kidney failure	0 (0.0)	5 (0.6)	0.1
Osteoporosis	58 (7.2)	45 (5.0)	0.06
Cancer	46 (5.7)	37 (4.1)	0.14
Gastro-oesophageal reflux disease	82 (10.2)	88 (9.7)	0.77
**Smoking history**	339 (42.3)	539 (59.4)	<0.001
Missing data	166 (20.7)	142 (15.6)	
**Weight (kg)**	53±12	67±15	<0.001
Missing data	124 (15.5)	90 (9.9)	
**Height (cm)**	160±7	173±7	<0.001
Missing data	122 (15.2)	90 (9.9)	
**BMI (kg·m^−2^)**	20.6±4.1	22.6±4.6	<0.001
Missing data	126 (15.7)	94 (10.4)	
**Pre-transplantation anti-HLA antibodies**	366 (45.6)	232 (25.6)	<0.001
Pre-transplantation anti-HLA class I antibodies			<0.001
None	518 (64.6)	739 (81.4)	
1–10	219 (27.3)	162 (17.8)	
11–20	33 (4.1)	3 (0.3)	
21–30	22 (2.7)	4 (0.4)	
>30	10 (1.2)	0 (0.0)	
Pre-transplantation anti-HLA class II antibodies			<0.001
None	571 (71.2)	775 (85.4)	
1–10	199 (24.8)	127 (14.0)	
11–20	31 (3.9)	5 (0.6)	
21–30	1 (0.1)	1 (0.1)	
**Bacterial colonisation**	375 (46.8)	491 (54.1)	0.01
Missing data	24 (3.0)	23 (2.5)	
**Fungal colonisation**	437 (54.5)	545 (60.0)	0.07
Missing data	24 (3.0)	23 (2.5)	
**6-min walk distance (% pred)**	45.3±16.4	43.1±17.0	0.026
Missing data	289 (36.0)	293 (32.3)	
**Non-invasive ventilation**	314 (39.2)	340 (37.4)	0.18
Missing data	77 (9.6)	69 (7.6)	
**Long-term oxygen**	651 (81.2)	779 (85.8)	0.035
Missing data	69 (8.6)	57 (6.3)	

Data are presented median (interquartile range), n (%) or mean±sd, unless otherwise stated. PH: pulmonary hypertension; BMI: body mass index; HLA: human leukocyte antigen. p-values estimated with the Chi-squared test, t-test or Wilcoxon test.

### Peri-transplantation phase

**FIGURE 3:**
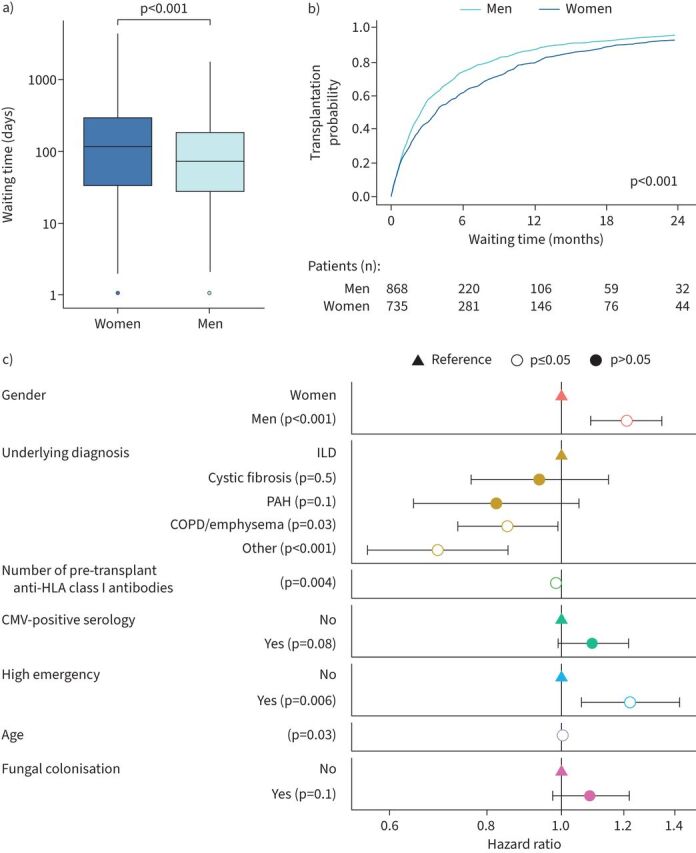
Waiting time before lung transplantation according to gender. a) Box plot representation of waiting time duration (log_10_ applied; Wilcoxon test). b) Kaplan–Meier transplantation curves in men and women over 24 months (log-rank test). c) Multivariable Cox proportional hazards model showing factors associated with a shorter or longer (hazard ratio >1 or <1, respectively) waiting time duration before lung transplantation. Variables with p<0.2 in the univariable analysis were selected and a forward stepwise selection using Akaike Information Criterion was used for the final multivariable Cox model. ILD: interstitial lung disease; PAH: pulmonary arterial hypertension; HLA: human leukocyte antigen; CMV: cytomegalovirus.

Lung transplantation was less often performed in women (735 out of 802 (91.7%)) than in men (868 out of 908 (95.6%); p=0.001). Reasons for removal from the waiting list were not significantly different according to gender (supplementary table S2). Time on the waiting list before lung transplantation differed markedly in women and men: women waited significantly longer before transplantation, with a median (IQR) of 115 (34–295) *versus* 73 (28–184) days for men (p<0.001) (table 2 and figure 3a and b). As for the procedure itself, women had less single lung transplantation (9.4% *versus* 15.8%; p<0.001) but more volume reduction than men (16.6% *versus* 7.8%; p<0.001) (table 2). We observed on univariable analysis that gender, age, height, underlying disease, positive anti-HLA antibodies pre-transplantation, specific listing on the high emergency programme, smoking history, cytomegalovirus-positive serology and 6-min walk test performance had a significant impact on waiting time duration before transplantation (supplementary table S3). Independent factors associated with a shorter waiting time duration before transplantation, identified in multivariable analysis, were male gender, age and listing on the high emergency lung transplantation programme. In contrast, the number of pre-transplantation class I anti-HLA antibodies was associated with an increased waiting time before transplantation, together with an underlying diagnosis of COPD and other underlying diseases (figure 3c and supplementary table S4).

**TABLE 2:** Peri-transplantation outcomes in women *versus* men lung transplant recipients

	Women (n=735)	Men (n=868)	p-value
**Waiting time before transplantation (days)**	115 (34–295)	73 (28–184)	<0.001
**High emergency**	124 (16.9)	117 (13.5)	0.07
**Procedure**			<0.001
Double lung	579 (78.8)	662 (76.3)	
Single lung	69 (9.4)	137 (15.8)	
Heart and lung	24 (3.3)	22 (2.5)	
Missing data	63 (8.6)	47 (5.4)	
**Volume reduction**	122 (16.6)	68 (7.8)	<0.001
Missing data	143 (19.5)	139 (16.0)	
**Total graft ischaemic time (min)**	603±165	609±168	0.51
**Gender mismatch**	279 (38.0)	242 (27.9)	<0.001
**Blood type mismatch**	42 (5.7)	51 (5.9)	0.94
Missing data	131 (17.8)	149 (17.2)	
**Weight mismatch^#^**			<0.001
No mismatch (−15– +15 kg)	344 (46.8)	437 (50.3)	
Donor weight > +15 kg recipient weight	303 (41.2)	266 (30.6)	
Donor weight < −15 kg recipient weight	31 (4.2)	115 (13.2)	
Missing data	57 (7.8)	50 (5.8)	
**Donor-to-recipient pTLC ratio**			<0.001
0.92–1.41	523 (71.2)	542 (62.4)	
<0.92	73 (9.9)	263 (30.3)	
>1.41	74 (10.1)	1 (0.1)	
Missing data	65 (8.8)	62 (7.1)	
**CMV mismatch**	140 (19.0)	181 (20.9)	0.55
Missing data	36 (4.9)	36 (4.1)	
**EBV mismatch**	48 (6.5)	52 (6.0)	0.54
Missing data	40 (5.4)	38 (4.4)	
**HLA incompatibilities (n)**	6 (5–6)	6 (5–7)	0.35
Missing data	72 (9.8)	77 (8.9)	
**Primary graft dysfunction**	184 (25.0)	185 (21.3)	0.2
Missing data	130 (17.7)	166 (19.1)	

Data are presented median (interquartile range), n (%) or mean±sd, unless otherwise stated. pTLC: predicted total lung capacity; CMV: cytomegalovirus; EBV: Epstein–Barr virus; HLA: human leukocyte antigen. ^#^: donor weight minus recipient weight, absolute value. p-values estimated with the Chi-squared test, t-test or Wilcoxon test.

Donor clinical characteristics are presented in supplementary table S5. A minority of recipients underwent gender-mismatched transplants, and women were more frequently transplanted with a male donor than men with a female donor (38.0% *versus* 27.9%; p<0.001) (table 2). More women than men had a high donor-to-recipient pTLC ratio (>1.41: 10.1% *versus* 0.1%; p<0.001). Additionally, more transplantations with lungs from a donor weighing >15 kg than the recipient were performed in women than in men (41.2% in women *versus* 30.6% in men; p<0.001). Similar findings were also observed using height and body surface area (data not shown). In summary, women waited longer to get a lung transplantation and gender was an independent factor associated with waiting list time. Lung volume mismatch with a donor of larger stature than the recipient was more frequently observed in women.

### Post-transplantation outcomes

**FIGURE 4:**
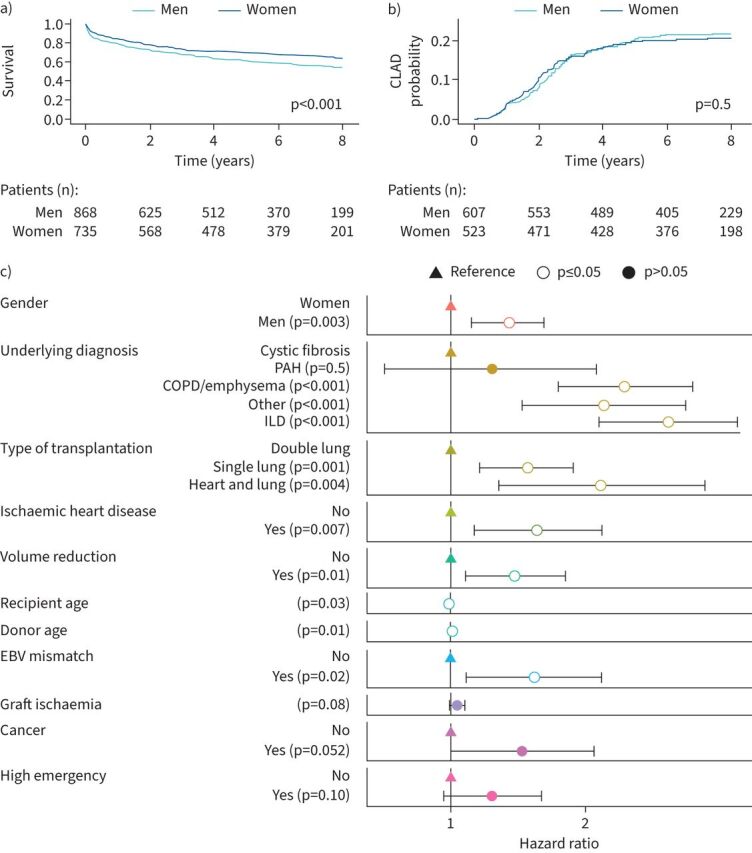
Overall survival and chronic lung allograft dysfunction (CLAD) occurrence following lung transplantation according to recipient gender. a) Kaplan–Meier survival curves over 8 years between women and men (log-rank test). b) Comparison of CLAD occurrence between women and men over 8 years (log-rank test). c) Multivariable Cox proportional hazards model showing factors associated with a shorter or longer (hazard ratio >1 or <1, respectively) survival. Variables with p<0.2 in the univariable analysis were selected and a forward stepwise selection using Akaike Information Criterion was used for the final multivariable Cox model. PAH: pulmonary arterial hypertension; ILD: interstitial lung disease; EBV: Epstein–Barr virus.

The median (IQR) follow-up post-transplantation for the entire cohort was 5.7 (1.9–7.9) years*.* After lung transplantation, survival was higher in women than in men, with death occurring in 34.4% of female recipients and 42.7% of male recipients (p<0.001) (table 3). In female recipients, survival at 1, 3 and 5 years post-transplantation was 83%, 73% and 70%, respectively, whereas in male recipients the figures were 79%, 69% and 61%, respectively (figure 4a). Main causes of death in both groups were infection and graft failure, with no significant gender difference (supplementary table S6). There was no significant gender difference in CLAD prevalence (14.7% of female recipients and 15.7% of male recipients; p=0.9), and time to CLAD did not differ significantly between both genders (figure 4b and table 3). Women and men also exhibited similar rates of acute cellular rejection (40.3% *versus* 39.0%; p=0.3) and antibody-mediated rejection (19.5% *versus* 17.9%; p=0.18). Finally, there was no difference in bacterial, fungal or viral infection occurrence, but cancer occurred more frequently after transplantation in men than women (12.3% *versus* 8.7%; p=0.02) (table 3).

**TABLE 3:** Post-transplantation outcomes in women *versus* men transplant recipients

	Women (n=735)	Men (n=868)	p-value
**Death**	253 (34.4)	371 (42.7)	<0.001
**Follow-up (years)**	6.1 (2.3–8.2)	5.3 (1.6–7.8)	0.001
**Patients with *k* acute cellular rejection episodes >grade A1 (per year)**			0.34
*k*=0	409 (55.6)	506 (58.3)	
*k*>0– ≤1	268 (36.5)	311 (35.8)	
*k*>1	28 (3.8)	28 (3.2)	
Missing data	30 (4.1)	23 (2.6)	
**Antibody-mediated rejection**	143 (19.5)	155 (17.9)	0.18
Missing data	30 (4.1)	23 (2.6)	
**Chronic lung allograft dysfunction**			0.88
No chronic lung allograft dysfunction	415 (56.5)	471 (54.3)	
Bronchiolitis obliterans syndrome	87 (11.8)	106 (12.2)	
Restrictive allograft syndrome	13 (1.8)	17 (2.0)	
Mixed	8 (1.1)	13 (1.5)	
Missing data	212 (28.8)	261 (30.1)	
**Cancer**	64 (8.7)	107 (12.3)	0.023
Missing data	30 (4.1)	23 (2.6)	
**Dialysis**	68 (9.3)	67 (7.7)	0.12
Missing data	85 (11.6)	79 (9.1)	
**Kidney transplantation**	10 (1.4)	10 (1.2)	0.26
Missing data	30 (4.1)	23 (2.6)	
**Post-transplantation anti-HLA donor-specific antibodies**	180 (24.5)	172 (19.8)	0.015
Missing data	30 (4.1)	23 (2.6)	
**Patients with *k* bacterial infection episodes (per year)**			0.06
*k*=0	158 (21.5)	207 (23.8)	
*k*>0– ≤1	448 (61.0)	495 (57.0)	
*k*>1– ≤2	55 (7.5)	66 (7.6)	
*k*>2	44 (6.0)	77 (8.9)	
Missing data	30 (4.1)	23 (2.6)	
**Patients with *k* fungal infection episodes (per year)**			0.2
*k*=0	383 (52.1)	454 (52.3)	
*k*>0– ≤1	283 (38.5)	329 (37.9)	
*k*>1	39 (5.3)	62 (7.1)	
Missing data	30 (4.1)	23 (2.6)	
**Patients with *k* viral infection episodes (per year)**			0.22
*k*=0	374 (50.9)	422 (48.6)	
*k*>0– ≤1	297 (40.4)	385 (44.4)	
*k* in>1	34 (4.6)	38 (4.4)	
Missing data	30 (4.1)	23 (2.6)	
**Re-transplant(s) during follow-up**	27 (3.7)	22 (2.5)	0.24

Data are presented n (%) or median (interquartile range), unless otherwise stated. HLA: human leukocyte antigen. p-values estimated with the Chi-squared test, t-test or Wilcoxon test.

Univariable analysis identified multiple variables as significantly associated with post-transplantation survival, including gender, age, donor age and donor-to-recipient pTLC ratio (supplementary table S7). Multivariable analysis identified that variables associated with a lower survival were male gender, single lung transplantation, heart and lung transplantation, volume reduction, history of ischaemic heart disease, COPD, ILD, other underlying diseases, Epstein–Barr virus mismatch, donor age and recipient age (figure 4c and supplementary table S8).

**FIGURE 5:**
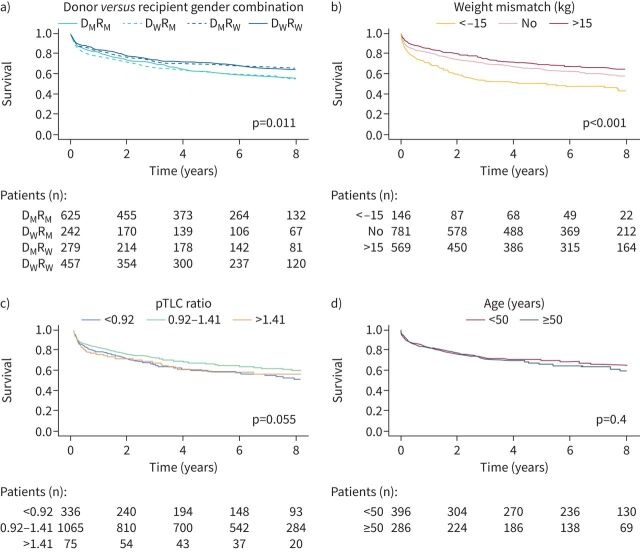
Factors influencing survival after lung transplantation. a) Survival curves according to donor *versus* recipient gender combinations over 8 years (log-rank test). D_M:_ man donor; D_W_: woman donor; R_M_: man recipient; R_W_: woman recipient. b) Survival curves according to weight mismatch (donor weight minus recipient weight). c) Survival curves according to donor-to-recipient predicted total lung capacity (pTLC) ratio. d) Survival curves according to age (in women recipients only).

Regarding gender matching combinations between donors and recipients, survival was not significantly different according to donor gender, in contrast to recipient gender, with a higher survival in female recipients even in cases of gender mismatch (p=0.01) (figure 5a). For the whole population, weight-negative mismatch (defined as donor weight minus recipient weight < −15 kg) had a negative impact on survival, whereas weight-positive mismatch (defined as donor weight−recipient weight >15 kg) did not impact survival (figure 5b). Similar results were observed when separating female and male recipients (supplementary figure S2). Survival rate according to pTLC ratio was similar between the three pTLC ratio categories (figure 5c). To question a possible role of menopause on survival in female recipients, we analysed survival according to age, and did not find a statistical difference between female recipients aged ≤50 and >50 years (p=0.4) (figure 5d). Overall, stature mismatch with high pTLC ratio or lower recipient weight compared to donor did not have any consequence on survival.

Finally, we performed a survival analysis between female and male recipients according to the underlying disease, showing a significantly lower survival for male recipients with COPD. No difference in survival according to gender was observed for other underlying diseases (supplementary figure S2).

## Discussion

Our study identified striking differences between men and women along their lung transplantation trajectory. Time on the waiting list before transplantation was on average 6 weeks longer for women, and fewer women were transplanted than men. The multivariable analysis identified, among other factors, female gender as an independent factor associated with a longer waiting time before transplantation. After transplantation, female recipient survival was significantly higher than that of male recipients, while the prevalence of specific lung transplantation outcomes, including primary graft dysfunction, CLAD, acute cellular rejection and infection, was not different according to gender.

One strength of our study lies in the multicentric, nationwide nature of the cohort, associating all existing centres in France and from a European setting, which presents differences with the north American context, notably in terms of recipient and donor characteristics [23, 24]. The specific reasons for which women waited longer before transplantation are complex to decipher but beyond the already known role of HLA sensitisation, we hypothesised that lung volume played an important role for delayed lung transplantation due to the lack of compatible donors. Gender disparities on lung transplant waiting lists have already been analysed in previous studies. The most recent and largest study also showed a longer median waiting list duration time for women [25], and another study by Wille *et al*. [10] showed that women were more likely than men to worsen or die within 3 years of registering on the waiting list in the USA (16% *versus* 11%; OR 1.58; p<0.001) and they were indeed less likely than men to be transplanted (83.9% *versus* 88.7%; OR 0.63; p<0.001). Sell *et al*. [11] also observed that short stature was associated with a lower rate of lung transplantation and higher rates of death and respiratory failure while awaiting transplantation, and that female patients were being disproportionately affected by this disparity. Transplantation with a lung volume mismatch may be more complex, and it has been shown that transplantation with an undersized lung (based on donor-to-recipient height ratio) was associated with worse post-transplantation outcomes [1, 22, 26–28]. Importantly, oversized transplanted lungs are not deleterious, except for candidates with ILD [1, 21, 22].

We observed that despite the fact that donors were mostly men (56%), taller by 16 cm and heavier by 22 kg compared to the average height and weight of female recipients, a majority of recipients were size-matched to their donor and most transplantations were performed with gender matching. However, when considering lung transplantation with lung volume mismatch, there were more female recipients than male recipients transplanted with a pTLC ratio >1.41, and in consequence more volume reduction in this group. Importantly, women overall who did receive an oversized lung did not have worse survival after transplantation than those who did not. Historically, size matching in France has relied upon height and sex only. This study may support a more widespread use of pTLC for size matching, which may be more accurate and may decrease the volume reduction numbers, as volume reduction was associated with reduced overall survival. As a result, it may possibly allow a higher proportion of female recipients to receive male donor transplants in a safe way, thus addressing some of the gender inequalities on waiting lists.

The other major known factor affecting waiting time before transplantation is pre-transplantation HLA immunisation. As pregnancy is an important trigger for immunisation, anti-HLA antibodies are mostly found in women. Several studies have highlighted this issue [29, 30]. Our work confirms these data and this result is a strength of our study; in the study discussed above, in which gender was associated with longer waiting time, this variable was not specifically assessed [11, 25].

The main limitation of our study, besides the retrospective nature of the analysis, lies in the cohort size, which is smaller compared to UNOS-based studies on this topic. Furthermore, some items presented a high rate of missing data, such as volume reduction or 6-min walk test data, and the unknown reasons for waiting list removal, which could not be retrieved.

Finally, increased late survival in female lung transplant recipients has been observed in several studies with different cohorts [1, 15, 28]. However, the reason for poorer overall survival in male recipients is equivocal and probably multifactorial. Indeed, men are significantly older than women at the time of lung transplantation and have more cardiovascular comorbidities. This is especially true for patients with COPD, in which even without lung transplantation, differences in survival between men and women are observed to the advantage of the latter [31]. This difference may also be partly related to factors that could also be gender related, including resumption of smoking after transplantation, poorer compliance or risk taking more frequently observed among men than women [32]. The fact that in our study gender difference in survival was only significant in COPD recipients, the only behavioural disease of concern here, supports this hypothesis. Another interesting result is that fewer women were listed for lung transplantation than men, which raises the question of unequal access to lung transplantation programmes. However, although women were younger, had less cardiovascular risk factors and were less frequently on long-term oxygen, we did not have information available to support the fact that listing criteria differed in the way that marginal men candidates were accepted for listing whereas women candidates were declined. Interestingly, we bring new evidence that CLAD incidence does not seem to be influenced by gender. Finally, Bonner *et al*. [23] recently analysed the intersection of race/ethnicity, gender and primary diagnosis on 1-year mortality and adjusted 5-year survival from UNOS data, identifying a clear impact of social factors.

### Conclusion

Women were less likely to be transplanted and waited significantly longer than men to get a lung transplant, and this longer duration for women may be related to shorter stature and the presence of pre-transplantation anti-HLA antibodies. This discrepancy was observed despite a higher number of volume reductions, to overcome more frequent oversizing in female recipients.

## Supplementary material

10.1183/23120541.00623-2024.Supp1 **Please note:** supplementary material is not edited by the Editorial Office, and is uploaded as it has been supplied by the author.
Supplementary material 00623-2024.SUPPLEMENT
