# “Increased delay to lung transplantation for women candidates: gender-based disparity matters in the lung transplant trajectory” Adrien Tissot, Anne-Sophie Coatanea, Olivia Rousseau, Antoine Roux, Benjamin Coiffard, Xavier Demant, Benjamin Renaud-Picard, Jérôme Le Pavec, Antoine Magnan, Jean-François Mornex, Thomas Villeneuve, Loïc Falque, Mathilde Salpin, Véronique Boussaud, Christiane Knoop, Martine Reynaud-Gaubert, Romain Kessler, Gaëlle Dauriat, David Lair, Aurore Foureau, François-Xavier Blanc, Mathilde Karakachoff, Patricia Lemarchand and the COLT consortium. *ERJ Open Res* 2025; 11: 00623-2024.

**DOI:** 10.1183/23120541.50623-2024

**Published:** 2025-09-08

This article was originally published with an error in figure 2. In the legend, the colours for the Men and Women groups had been inverted. This error does not affect the conclusions of the article. The corrected figure is shown below. This has also been corrected in the article itself.

**FIGURE 2:**
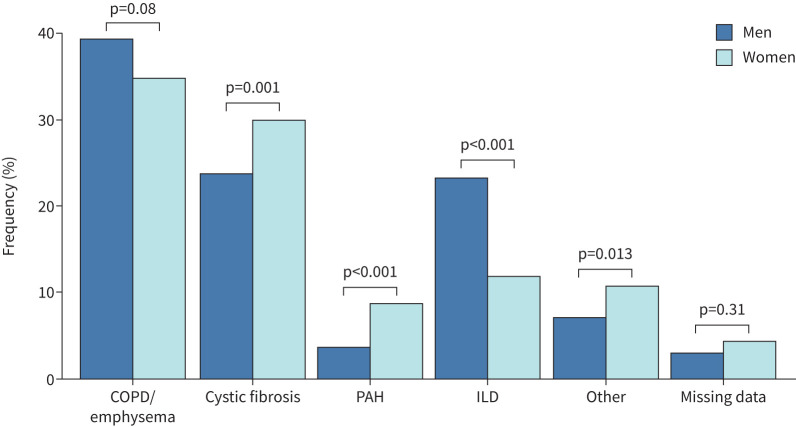
Underlying diagnosis in women and men awaiting lung transplantation. PAH: pulmonary arterial hypertension; ILD: interstitial lung disease. Chi-squared test used.

