# Supplementary material for: Increased delay to lung transplantation for women candidates: gender-based disparity matters in the lung transplant trajectory
Source: ERJ Open Res. 2025 May 6;11(3):00623-2024. doi: 10.1183/23120541.00623-2024 (PMC12053738; doi:10.1183/23120541.00623-2024)
Supplement: Supplementary file 1 [file 00623-2024.SUPPLEMENT.pdf]

Supplementary Appendix

Supplemental 1. French lung allocation policy 2
Supplemental table 1. Pulmonary function tests at registration on waiting list in female versus male patients with cystic fibrosis, ILD, COPD and pulmonary arterial hypertension. 3
Supplemental table 2. Causes of removal from waiting list 4
Supplemental table 3. Univariate Cox regression analysis for waiting time duration before lung transplantation and gender-adjusted analysis. 5
Supplemental table 4. Multivariable Cox regression analysis for waiting time duration before lung transplantation. 8
Supplemental table 5. Clinical characteristics of female and male donors 9
Supplemental table 6. Cause of death after transplantation 10
Supplemental table 7. Univariate Cox regression for overall survival, and gender-adjusted analysis 11
Supplemental table 8. Multivariable Cox regression for overall survival 16
Supplemental figure 1. Comparison of survival between women and men over 8 years according to underlying diagnosis. 17
Supplemental figure 2. Comparison of survival according to weight mismatch (donor weight – recipient weight) over 8 years. 18
Supplemental figure 3. pTLC ratio nonlinear association with declining risk of death. 19

Supplemental 1. French lung allocation policy

Lung transplant candidates whose life is threatened in the very short term are given a priority access at the national level, according to procedures and terms defined by the Agence de la Biomédecine which includes a referral to a college of experts. If several recipients are registered in the lung high-emergency category, the graft is offered to the earliest candidate registered in this category. Priority is granted for 8 days and may be extended to another 8 days.

The next level of allocation is local, in which the donor lung is proposed to the nearest lung transplantation centre from the donor site.

The final level of allocation is a national round where the donor lung is proposed successively to the lung transplantation teams.

Apart from the high-emergency procedure, the choice of the recipient within its waiting list is the responsibility of the lung transplantation team.

Overall, donor proposition is made for a candidate of similar blood group. Special access for a different compatible group can be asked to the agency with expert review (e.g. B candidate for O donor).

Supplemental table 1. Pulmonary function tests at registration on waiting list in female versus male patients with cystic fibrosis, ILD, COPD and pulmonary arterial hypertension.

Variables	Women	Men	p-value[a]
**Cystic fibrosis**	**n = 242**	**n = 214**	
FEV1 as % of predicted	24.0 [19.9 ; 29.0]	22.0 [19.0 ; 27.2]	0.014
Missing data (%)	9 (3.7)	5 (2.3)	
FVC as % of predicted	41.0 [33.0 ; 50.0]	41.0 [33.0 ; 48.1]	0.26
Missing data (%)	14 (5.8)	10 (4.7)	
DLCO as % of predicted	46.0 [37.5 ; 53.4]	46 [38 ; 56]	0.73
Missing data (%)	199 (82.2)	170 (79.4)	
TLC as % of predicted	105 [95 ; 117]	102 [87 ; 114]	0.08
Missing data (%)	112 (46.3)	97 (45.3)	
**Interstitial lung disease**	**n = 96**	**n = 209**	
FEV1 as % of predicted	42.5 [33.8 ; 54.0]	51.0 [38.1 ; 61.0]	0.001
Missing data (%)	12 (12.5)	11 (5.3)	
FVC as % of predicted	40 [33 ; 50]	46 [36 ; 58]	0.002
Missing data (%)	11 (11.5)	14 (6.7)	
DLCO as % of predicted	25.6 [18.4 ; 34.8]	22.0 [17.0 ; 30.0]	0.14
Missing data (%)	70 (72.9)	89 (42.6)	
TLC as % of predicted	47 [41 ; 61]	50 [43 ; 62]	0.32
Missing data (%)	39 (40.6)	51 (24.4)	
**COPD/emphysema**	**n = 279**	**n = 358**	
FEV1 as % of predicted	22.0 [18.0 ; 27.0]	20.5 [17.0 ; 27.0]	0.12
Missing data (%)	12 (4.3)	17 (4.7)	
FVC as % of predicted	56.4 [43.0 ; 70.0]	54.0 [44.0 ; 69.0]	0.59
Missing data (%)	16 (5.7)	26 (7.3)	
DLCO as % of predicted	20.0 [13.4 ; 27.6]	22.0 [16.0 ; 29.9]	0.17
Missing data (%)	161 (57.7)	184 (51.4)	
TLC as % of predicted	139 [126 ; 155]	125 [110 ; 140]	<0.001
Missing data (%)	80 (28.7)	94 (26.3)	
**Pulmonary arterial hypertension**	**n = 67**	**n = 34**	
FEV1 as % of predicted	78 [65 ; 89]	83.0 [62.3 ; 97.2]	0.55
Missing data (%)	14 (20.9)	6 (17.6)	
FVC as % of predicted	94 [76 ; 104]	94 [70 ; 102]	0.6
Missing data (%)	17 (25.4)	8 (23.5)	
DLCO as % of predicted	58.0 [40.2 ; 66.3]	35.0 [27.8 ; 57.6]	0.016
Missing data (%)	35 (52.2)	12 (35.3)	
TLC as % of predicted	94 [84 ; 108]	93 [82 ; 100]	0.47
Missing data (%)	23 (34.3)	9 (26.5)	

Data are expressed as median [interquartile range] and missing data as number (%).
[a] Estimated using the Wilcoxon test. FEV1: forced expiratory volume in one second. FVC, forced vital capacity; DLCO, diffusing capacity for carbon monoxide; TLC, total lung capacity; COPD, chronic obstructive pulmonary disease.

Supplemental table 2. Causes of removal from waiting list

	Women (n = 66)	Men (n = 41)	p-value
**Cause (%)**			0.07
**Improvement**	5 (7.7)	0 (0.0)	
**Contra indication**	4 (6.2)	5 (12.2)	
**Death**	34 (52.3)	28 (68.3)	
**Undetermined**	23 (33.8)	8 (19.5)	

Supplemental table 3. Univariate Cox regression analysis for waiting time duration before lung transplantation and gender-adjusted analysis.

Variables	Univariate analysis		Gender adjusted analysis (Surv(delay, transplantation) ~ gender + variable)[a]			
	HR (95% CI)	P-value	Gender adjusted HR (95% CI)	Gender adjusted p-value	Variable adjusted HR (95% CI)	Variable adjusted p-value
**Male gender**	1.28 (1.16-1.42)	<0.001				
**Age**	1.01 (1.00-1.01)	0.001	1.26 (1.14-1.39)	<0.001 ***	1.00 (1.00-1.01)	0.006 *
**Underlying diagnosis**						
Interstitial lung disease	Ref.	-	1.25 (1.13-1.38)	<0.001 ***	Ref.	-
Cystic fibrosis	0.76 (0.66-0.88)	<0.001	-	-	0.78 (0.67-0.91)	0.001 *
Pulmonary arterial hypertension	0.77 (0.61-0.97)	0.029	-	-	0.81 (0.64-1.02)	0.075
COPD/emphysema	0.83 (0.72-0.95)	0.008	-	-	0.83 (0.72-0.96)	0.012
Other	0.68 (0.55-0.83)	<0.001	-	-	0.69 (0.57-0.85)	<0.001 *
**CMV positive serology**	1.12 (1.01-1.24)	0.026	1.28 (1.16-1.42)	<0.001 ***	1.13 (1.02-1.25)	0.016
EBV positive serology	0.99 (0.82-1.21)	0.942	1.29 (1.16-1.43)	<0.001 ***	0.98 (0.81-1.19)	0.838
Bacterial colonization	1.01 (0.91-1.11)	0.903	1.28 (1.16-1.41)	<0.001 ***	0.99 (0.90-1.10)	0.921
**Fungal colonization**	1.10 (1.00-1.22)	0.058	1.27 (1.15-1.41)	<0.001 ***	1.09 (0.98-1.20)	0.103
**6-minute walk test, % of predicted**	1.00 (0.99-1.00)	0.005	1.31 (1.16-1.47)	<0.001 ***	1.00 (0.99-1.00)	0.026
Non-invasive ventilation	0.98 (0.88-	0.692	1.26 (1.13-1.39)	<0.001 ***	0.98 (0.88-1.09)	0.711

	1.09)					
**Long-term oxygen**	1.14 (0.96-1.36)	0.14	1.27 (1.14-1.40)	<0.001 ***	1.14 (0.96-1.35)	0.149
Ischemic heart disease	1.11 (0.88-1.39)	0.391	1.28 (1.16-1.42)	<0.001 ***	1.05 (0.83-1.32)	0.698
High blood pressure	1.10 (0.94-1.29)	0.218	1.28 (1.16-1.41)	<0.001 ***	1.06 (0.91-1.24)	0.448
**Pulmonary hypertension group III**	0.86 (0.73-1.01)	0.06	1.29 (1.16-1.42)	<0.001 ***	0.86 (0.73-1.00)	0.054
Dyslipidemia	1.10 (0.90-1.36)	0.35	1.28 (1.16-1.42)	<0.001 ***	1.06 (0.86-1.31)	0.58
Diabetes	0.94 (0.82-1.07)	0.349	1.28 (1.16-1.42)	<0.001 ***	0.94 (0.82-1.08)	0.393
Heart failure	0.86 (0.54-1.36)	0.516	1.28 (1.16-1.42)	<0.001 ***	0.84 (0.53-1.34)	0.476
Gastroesophageal reflux	0.98 (0.83-1.15)	0.786	1.28 (1.16-1.42)	<0.001 ***	0.97 (0.82-1.14)	0.713
Kidney failure	1.76 (0.56-5.45)	0.331	1.28 (1.16-1.42)	<0.001 ***	1.57 (0.50-4.88)	0.436
Cancer	1.00 (0.80-1.26)	0.996	1.28 (1.16-1.42)	<0.001 ***	1.00 (0.80-1.26)	0.993
**Number of pre-transplant anti-HLA class I antibodies**	0.98 (0.97-0.99)	<0.001	1.25 (1.13-1.38)	<0.001 ***	0.99 (0.98-1.00)	0.005 *
**Number of pre-transplant anti-HLA class II antibodies**	0.98 (0.96-0.99)	0.01	1.26 (1.14-1.40)	<0.001 ***	0.98 (0.96-1.00)	0.072
**Height, cm**	1.01 (1.01-1.02)	<0.001	1.26 (1.10-1.45)	0.001 **	1.00 (1.00-1.01)	0.505
Recipient's blood type						
A	Ref.	-	1.32 (1.19-1.48)	<0.001 ***	Ref.	-
AB	1.08 (0.81-1.45)	0.586	-	-	1.06 (0.79-1.43)	0.674
B	1.10 (0.92-	0.308	-	-	1.09 (0.91-1.30)	0.371

	1.32)					
O	0.94 (0.84-1.06)	0.296	-	-	0.91 (0.81-1.03)	0.131
**Smoking history**	0.90 (0.80-1.01)	0.061	1.27 (1.14-1.42)	<0.001 ***	0.87 (0.78-0.97)	0.016
**High emergency**	1.14 (1.00-1.31)	0.057	1.30 (1.18-1.43)	<0.001 ***	1.18 (1.03-1.36)	0.018

[a] Where Surv() is the survival function; delay is the time between the day of registration on the waiting list and transplantation (or the day of data extraction for non-transplanted patients), transplantation is the event of the survival analysis, gender is present in all the adjusted bivariable analysis, and variable corresponds to the other explanatory variables. Results with significant p-values corrected by Benjamini-Hochberg are indicated as follows: * $p < 0.05$, ** $p < 0.01$, and *** $p < 0.001$. HR, hazard ratio; CI, confidence interval; COPD, chronic obstructive pulmonary disease; CMV, Cytomegalovirus; EBV, Epstein-Barr Virus; HLA, human leukocyte antigen.

Supplemental table 4. Multivariable Cox regression analysis for waiting time duration before lung transplantation.

Variables (n = 1,529)	HR (95% CI)	P-value
Male gender	1.21 [1.09-1.34]	<0.001
Underlying diagnosis		
Interstitial lung disease	Ref.	-
Cystic fibrosis	0.94 [0.77-1.15]	0.52
Pulmonary arterial hypertension	0.82 [0.64-1.05]	0.12
COPD/emphysema	0.85 [0.73-0.99]	0.03
Other	0.69 [0.56-0.85]	<0.001
Number of pre-transplant anti-HLA class I antibodies	0.98 [0.97-1.00]	<0.001
CMV positive serology	1.10 [0.99-1.22]	0.08
High-emergency	1.23 [1.06-1.42]	0.01
Recipient age	1.01 [1.00-1.01]	0.03
Fungal colonization	1.09 [0.97-1.22]	0.13

HR, hazard ratio; CI, confidence interval; COPD, chronic obstructive pulmonary disease; HLA, human leukocyte antigen; CMV, Cytomegalovirus.

Supplemental table 5. Clinical characteristics of female and male donors

Variables	Women (n = 699)	Men (n = 904)	p-value[a]
Age, years	51.0 [40.0 ; 59.0]	44.5 [30.0 ; 56.0]	<0.001
Missing data (%)[b]	55 (7.9)	56 (6.2)	
Weight, kg	65 (± 13)	75 (± 13)	<0.001
Height, cm	163 (± 7)	176 (± 7)	<0.001
Body mass index, kg/m2	24.3 (± 4.7)	24.2 (± 3.6)	0.69
Cause of death			<0.001
Vascular	453 (64.8)	390 (43.1)	
Traumatic, public road accident	59 (8.4)	154 (17.0)	
Traumatic, non-road accident	61 (8.7)	184 (20.4)	
Anoxia	98 (14.0)	139 (15.4)	
Meningitis	11 (1.6)	11 (1.2)	
Tumor	8 (1.1)	2 (0.2)	
Intoxication	3 (0.4)	5 (0.6)	
Other	6 (0.9)	18 (2.0)	
Missing data (%)	0 (0.0)	1 (0.1)	
Smoking history	244 (34.9)	377 (41.7)	0.007
Blood type			0.41
A	273 (39.1)	329 (36.4)	
AB	12 (1.7)	20 (2.2)	
B	60 (8.6)	82 (9.1)	
O	298 (42.6)	416 (46.0)	
Missing data (%)	56 (8.0)	57 (6.3)	
CMV positive serology	369 (52.8)	420 (46.5)	0.014
EBV positive serology	678 (97.0)	852 (94.2)	0.027
Missing data (%)	0 (0.0)	1 (0.1)	
Toxoplasmosis positive serology	355 (50.8)	495 (54.8)	0.27
Missing data (%)	98 (14.0)	112 (12.4)	

Data are expressed as numbers (%), means (± standard deviations), or medians [interquartile ranges]. [a]Estimated with $\chi 2$, Student's, or Wilcoxon tests. [b] There are no missing data for variables for which there is no corresponding line. CMV, Cytomegalovirus; EBV, Epstein-Barr Virus.

Supplemental table 6. Cause of death after transplantation

	Women (n = 253)	Men (n = 371)	p-value
**Cause of death (%)**			0.51
**Cancer**	15 (5.9)	28 (7.5)	
**Cardiovascular**	19 (7.5)	23 (6.2)	
**Cerebrovascular**	4 (1.6)	8 (2.2)	
**Digestive**	0 (0.0)	1 (0.3)	
**Graft failure**	91 (36.0)	116 (31.3)	
**Hemorrhage**	23 (9.1)	23 (6.2)	
**Infection**	58 (22.9)	95 (25.6)	
**MOF**	14 (5.5)	29 (7.8)	
**Other**	13 (5.1)	15 (4.0)	
**Traumatic**	0 (0.0)	3 (0.8)	
**Unknown**	16 (6.3)	30 (8.1)	

Supplemental table 7. Univariate Cox regression for overall survival, and gender-adjusted analysis

Variables	Univariate analysis		Gender adjusted analysis (Surv(time, rejection) ~ gender + variable)[a]			
	HR (95% CI)	P-value	Gender adjusted HR (95% CI)	Gender adjusted p-value	Variable adjusted HR (95% CI)	Variable adjusted p-value
Male gender	1.32 (1.12-1.55)	0.001				
Age	1.01 (1.01-1.02)	<0.001	1.26 (1.07-1.48)	0.006 **	1.01 (1.01-1.02)	<0.001 ***
Underlying diagnosis						
Cystic fibrosis	Ref.	-	1.19 (1.01-1.41)	0.035 *	Ref.	-
Pulmonary arterial hypertension	1.32 (0.88-1.98)	0.177	-	-	1.35 (0.90-2.03)	0.147
COPD/emphysema	1.82 (1.46-2.26)	<0.001	-	-	1.80 (1.45-2.24)	<0.001 ***
Other	2.04 (1.50-2.78)	<0.001	-	-	2.08 (1.52-2.83)	<0.001 ***
Interstitial lung disease	2.82 (2.23-3.58)	<0.001	-	-	2.72 (2.14-3.46)	<0.001 ***
CMV positive serology	1.07 (0.91-1.25)	0.431	1.32 (1.12-1.55)	0.001 **	1.08 (0.92-1.26)	0.362
EBV positive serology	0.84 (0.63-1.13)	0.254	1.32 (1.12-1.55)	0.001 **	0.84 (0.63-1.12)	0.235
Bacterial colonization	1.40 (1.20-1.65)	<0.001	1.28 (1.09-1.51)	0.002 **	1.39 (1.18-1.62)	<0.001 **
Fungal colonization	1.11 (0.94-1.30)	0.209	1.30 (1.11-1.53)	0.001 **	1.10 (0.94-1.29)	0.248
6-minute walk test, % of predicted	1.00 (0.99-1.00)	0.096	1.40 (1.15-1.70)	0.001 **	1.00 (0.99-1.00)	0.147
On noninvasive ventilation before transplantation	0.75 (0.63-0.88)	0.001	1.31 (1.11-1.55)	0.001 *	0.74 (0.63-0.88)	0.001 **

Long term oxygen supplementation	1.07 (0.80-1.41)	0.657	1.32 (1.12-1.56)	0.001 **	1.04 (0.78-1.37)	0.808
Ischemic heart disease	1.87 (1.39-2.52)	<0.001	1.29 (1.10-1.52)	0.002 **	1.80 (1.34-2.43)	<0.001 **
High blood pressure	1.12 (0.88-1.42)	0.367	1.31 (1.12-1.54)	0.001 **	1.09 (0.86-1.39)	0.483
Secondary pulmonary arterial hypertension	0.95 (0.73-1.22)	0.669	1.32 (1.12-1.55)	0.001 **	0.94 (0.73-1.21)	0.642
Dyslipidemia	1.51 (1.13-2.02)	0.006	1.30 (1.11-1.53)	0.001 **	1.46 (1.09-1.96)	0.011 *
Diabetes	0.78 (0.62-0.98)	0.033	1.32 (1.13-1.55)	0.001 **	0.77 (0.61-0.97)	0.025
Heart failure	1.15 (0.57-2.30)	0.701	1.32 (1.12-1.54)	0.001 **	1.13 (0.56-2.27)	0.735
Gastroesophageal reflux	0.77 (0.58-1.03)	0.075	1.32 (1.12-1.55)	0.001 **	0.77 (0.58-1.02)	0.073
Kidney failure	0.00 (0.00-Inf)	0.988	1.32 (1.13-1.55)	0.001 **	0.00 (0.00-Inf)	0.988
Osteoporosis	0.96 (0.69-1.34)	0.827	1.32 (1.12-1.54)	0.001 **	0.98 (0.70-1.37)	0.906
Cancer	1.60 (1.17-2.19)	0.003	1.33 (1.13-1.56)	0.001 **	1.63 (1.19-2.23)	0.002 *
Pre-transplant anti-HLA antibodies	0.88 (0.75-1.05)	0.149	1.30 (1.10-1.53)	0.002 **	0.93 (0.78-1.10)	0.413
Height, cm	1.01 (1.00-1.02)	0.085	1.36 (1.10-1.68)	0.005 **	1.00 (0.99-1.01)	0.59
pTLC ratio between donor and recipient						
[0.92 ; 1.41]	Ref.	-	1.30 (1.09-1.55)	0.003 **	Ref.	-
< 0.92	1.25 (1.04-1.51)	0.02	-	-	1.17 (0.96-1.41)	0.116
> 1.41	1.19 (0.83-1.70)	0.346	-	-	1.36 (0.94-1.98)	0.104

Recipient's blood type						
A	Ref.	-	1.31 (1.10-1.55)	0.002 **	Ref.	-
AB	0.93 (0.58-1.49)	0.768	-	-	0.92 (0.58-1.47)	0.729
B	0.91 (0.68-1.21)	0.517	-	-	0.92 (0.69-1.23)	0.578
O	0.96 (0.80-1.15)	0.661	-	-	0.96 (0.80-1.15)	0.636
Smoking history	1.53 (1.26-1.85)	<0.001	1.33 (1.11-1.61)	0.002 **	1.46 (1.20-1.77)	<0.001 **
High emergency	1.47 (1.21-1.80)	<0.001	1.33 (1.13-1.56)	0.001 **	1.49 (1.22-1.82)	<0.001 **
Type of transplantation						
Double lung transplant	Ref.	-	1.28 (1.08-1.51)	0.004 **	Ref.	-
Single lung transplant	1.72 (1.14-2.60)	0.01	-	-	1.76 (1.16-2.65)	0.007 *
Heart and lung transplant	1.87 (1.53-2.29)	<0.001	-	-	1.81 (1.48-2.21)	<0.001 ***
Volume reduction	1.24 (0.98-1.57)	0.068	1.45 (1.21-1.73)	<0.001 **	1.35 (1.07-1.71)	0.013 *
Graft ischemia, hours	1.04 (1.01-1.07)	0.012	1.31 (1.12-1.54)	0.001 **	1.04 (1.01-1.07)	0.016
Gender mismatch	0.97 (0.82-1.15)	0.745	1.32 (1.12-1.55)	0.001 **	1.00 (0.85-1.19)	0.962
Blood type mismatch	0.95 (0.68-1.33)	0.755	1.31 (1.10-1.55)	0.002 **	0.95 (0.68-1.33)	0.757
Weight mismatch (D-R)						
No weight mismatch	Ref.	-	1.23 (1.04-1.45)	0.016 *	Ref.	-
Donor weight < -15 kg recipient weight	1.57 (1.22-2.01)	<0.001	-	-	1.50 (1.17-1.92)	0.002 **
Donor weight > +15 kg recipient weight	0.83 (0.70-	0.038	-	-	0.84 (0.71-1.01)	0.061

	0.99)					
CMV mismatch	1.08 (0.89-1.31)	0.418	1.31 (1.12-1.54)	0.001 **	1.08 (0.89-1.31)	0.432
EBV mismatch	1.26 (0.94-1.70)	0.122	1.32 (1.12-1.55)	0.001 **	1.27 (0.94-1.70)	0.114
Count of HLA incompatibilities	1.00 (0.94-1.06)	0.963	1.30 (1.10-1.54)	0.002 **	1.00 (0.94-1.06)	0.936
Primary graft dysfunction	1.56 (1.29-1.89)	<0.001	1.38 (1.15-1.65)	0.001 **	1.60 (1.32-1.93)	<0.001 ***
Donor age, years	1.01 (1.00-1.01)	0.002	1.32 (1.12-1.56)	0.001 **	1.01 (1.00-1.01)	0.001 **
Weight, kg	1.00 (0.99-1.00)	0.648	1.35 (1.15-1.60)	<0.001 **	1.00 (0.99-1.00)	0.179
Height, cm	1.00 (0.99-1.01)	0.707	1.40 (1.18-1.66)	<0.001 **	0.99 (0.98-1.00)	0.073
Body mass index, kg/m2	1.00 (0.98-1.02)	0.818	1.32 (1.12-1.55)	0.001 **	1.00 (0.98-1.02)	0.707
Cause of death						
Vascular	Ref.	-	1.32 (1.12-1.55)	0.001 **	Ref.	-
Traumatic, public road accident	0.89 (0.70-1.14)	0.354	-	-	0.88 (0.69-1.13)	0.307
Traumatic, non-road accident	1.07 (0.85-1.33)	0.58	-	-	1.04 (0.83-1.31)	0.717
Anoxia	0.99 (0.79-1.25)	0.951	-	-	0.98 (0.78-1.24)	0.886
Meningitis	1.57 (0.88-2.80)	0.124	-	-	1.58 (0.89-2.82)	0.117
Tumor	0.66 (0.21-2.07)	0.478	-	-	0.62 (0.20-1.95)	0.416
Intoxication	2.07 (0.86-5.01)	0.106	-	-	1.92 (0.79-4.65)	0.149

Other	0.89 (0.46-1.72)	0.728	-	-	0.84 (0.43-1.63)	0.608
Smoking history	1.02 (0.87-1.20)	0.801	1.32 (1.12-1.54)	0.001 **	1.01 (0.86-1.18)	0.936
Blood type						
A	Ref.	-	1.30 (1.10-1.53)	0.002 **	Ref.	-
AB	0.88 (0.48-1.61)	0.671	-	-	0.86 (0.47-1.57)	0.62
B	1.06 (0.80-1.42)	0.667	-	-	1.08 (0.81-1.44)	0.594
O	1.00 (0.84-1.19)	0.989	-	-	1.00 (0.84-1.19)	0.996
Anti-CMV antibodies	1.08 (0.92-1.26)	0.363	1.32 (1.13-1.55)	0.001 **	1.09 (0.93-1.27)	0.293
Anti-EBV antibodies	1.09 (0.74-1.60)	0.662	1.32 (1.12-1.55)	0.001 **	1.11 (0.75-1.63)	0.609
Anti-toxoplasmosis antibodies	1.03 (0.87-1.23)	0.734	1.33 (1.12-1.58)	0.001 **	1.03 (0.87-1.23)	0.724

[a] Where *Surv()* is the survival function, *time* is the time between the transplantation day and the rejection or the day of the last follow-up, *rejection* is the event of the survival analysis, *gender* is present in all the adjusted bivariable analyses, *variable* corresponds to the other explanatory variables.
Results with significant p-values corrected by Benjamini-Hochberg are indicated as follows: * p < 0.05, ** p < 0.01, and *** p < 0.001. HR, hazard ratio; CI, confidence interval; COPD, chronic obstructive pulmonary disease; CMV, Cytomegalovirus; EBV, Epstein-Barr Virus; pTLC, predicted total lung capacity; HLA, human leukocyte antigen; D: Donor, DSA, donor-specific antibodies; R: Recipient.

Supplemental table 8. Multivariable Cox regression for overall survival

Variables (n = 1,256)	HR (95% CI)	P-value
Male gender	1.34 [1.11-1.62]	<0.001
Underlying diagnosis		
Cystic fibrosis	Ref.	-
Pulmonary arterial hypertension	1.23 [0.71-2.12]	0.47
COPD/emphysema	2.46 [1.74-3.48]	<0.001
Other	2.21 [1.45-3.35]	<0.001
Interstitial lung disease	3.09 [2.14-4.45]	<0.001
Type of transplantation		
Double lung transplant	Ref.	-
Single lung transplant	1.48 [1.16-1.89]	<0.001
Heart and lung transplant	2.18 [1.28-3.69]	<0.001
Ischemic heart disease	1.57 [1.13-2.17]	0.01
Volume reduction	1.39 [1.07-1.81]	0.01
Recipient age	0.99 [0.98-1.00]	0.03
Donor age	1.01 [1.00-1.01]	0.01
EBV mismatch	1.54 [1.09-2.17]	0.02
Graft ischemia, hours	1.03 [1.00-1.07]	0.08
Cancer	1.44 [1.00-2.09]	0.05
High emergency	1.24 [0.96-1.59]	0.1

After the pre-selection of variables with a p-value < 0.2 in the univariate analysis, a stepwise forward selection was applied with the Akaike information criterion to create a multivariate model with the recipient's gender. HR, hazard ratio; CI, confidence interval; COPD, chronic obstructive pulmonary disease; EBV, Epstein-Barr Virus.

Supplemental figure 1. Comparison of survival between women and men over 8 years according to underlying diagnosis.

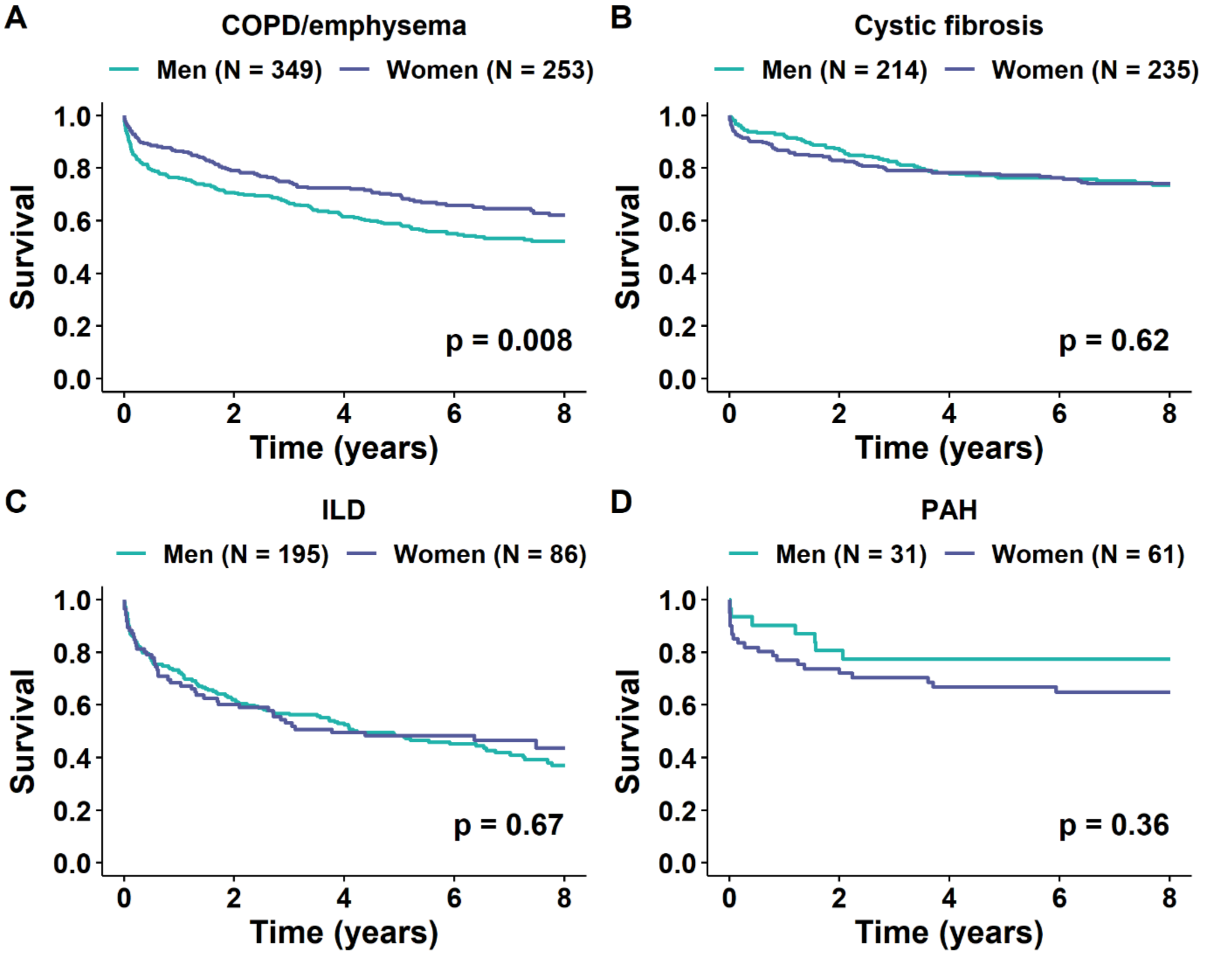

Log-rank tests used. (A) COPD/emphysema. COPD: Chronic obstructive pulmonary disease. (B) Cystic fibrosis. (C) ILD: Interstitial lung disease. (D) PAH: Pulmonary arterial hypertension.

Supplemental figure 2. Comparison of survival according to weight mismatch (donor weight – recipient weight) over 8 years.

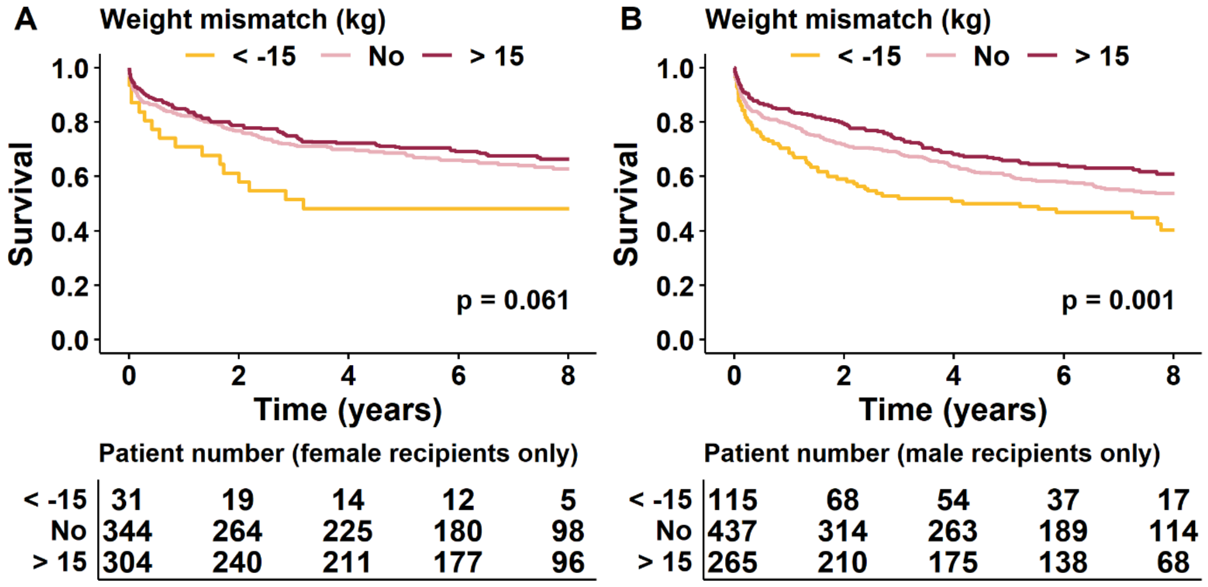

Log-rank tests used. *No* corresponds to no mismatch (with a weight difference between -15 and 15kg). (A) Only female recipients and (B) only male recipients are considered.

Supplemental figure 3. pTLC ratio nonlinear association with declining risk of death.

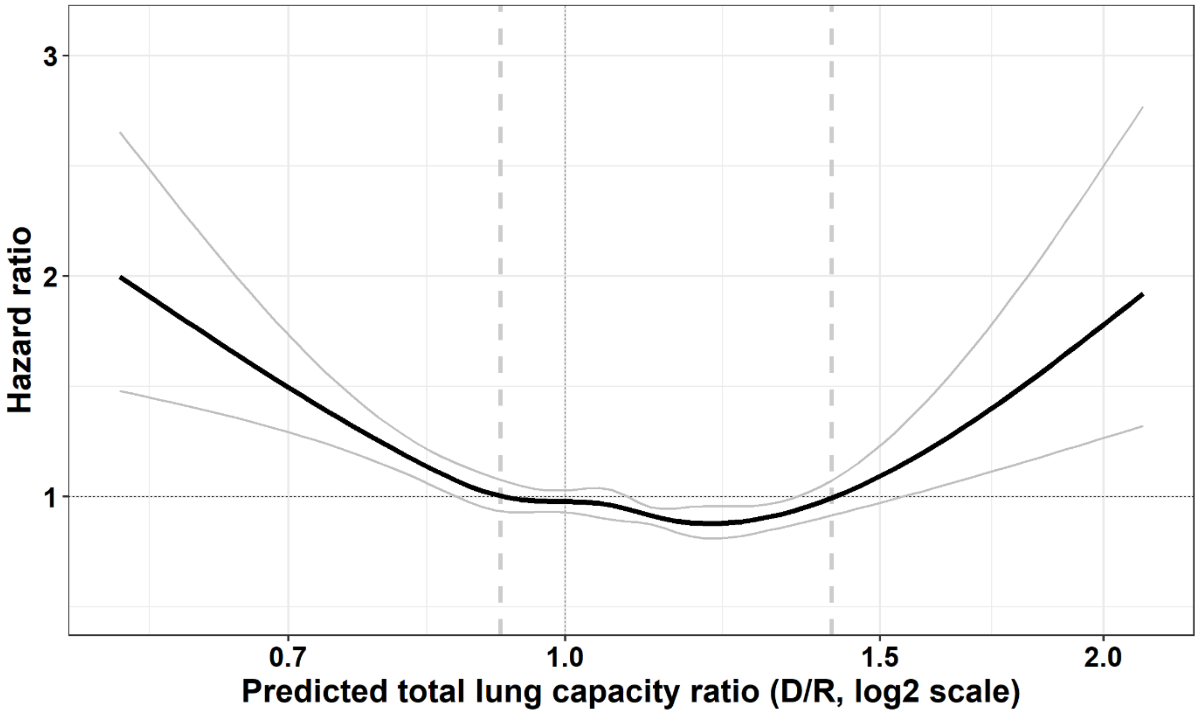

The impact of pTLC ratio values on recipient survival was computed with a Cox model. pTLC ratios were modelized with a spline to deal with the nonlinear relationship between those variables. The intersection points between pTLC ratio spline with the hazard ratio value 1 served as cutoff points to categorize pTLC ratio values.
